# Elastin in the Pathogenesis of Abdominal Aortic Aneurysm

**DOI:** 10.3390/cells14201597

**Published:** 2025-10-14

**Authors:** Dunpeng Cai, Shi-You Chen

**Affiliations:** 1 Departments of Surgery, University of Missouri School of Medicine, Columbia, MO 65212, USA; dccfn@health.missouri.edu; 2 The Research Service, Harry S. Truman Memorial Veterans Hospital, Columbia, MO 65201, USA

**Keywords:** elastin, abdominal aortic aneurysm (AAA), extracellular matrix (ECM), vascular smooth muscle cells, matrix metalloproteinases, tissue inhibitors of matrix metalloproteinases

## Abstract

Abdominal aortic aneurysms (AAAs) are progressive, life-threatening vascular disorders characterized by focal dilation of the abdominal aorta due to chronic weakening of the arterial wall. The condition often remains asymptomatic until rupture, which carries mortality rates exceeding 70–85%. Among the various etiological theories of AAA development, degradation of the extracellular matrix (ECM) has emerged as the most widely accepted paradigm, with the breakdown of elastin representing a central and irreversible hallmark event. Elastin, a highly cross-linked and durable structural protein, provides elasticity and recoil to the aortic wall. In human AAA specimens, reduced elastin content, impaired cross-linking, and extensive fiber fragmentation are consistently observed, while experimental studies across multiple animal models confirm that elastin degradation directly correlates with aneurysm initiation, expansion, and rupture risk. Elastin loss is driven by a complex interplay of proteolytic enzymes coupled with inflammatory cell infiltration and oxidative stress. Furthermore, elastin-derived peptides perpetuate immune cell recruitment and matrix degradation, creating a vicious cycle of wall injury. Genetic and epigenetic factors, including variants in ECM regulators and dysregulation of non-coding RNAs, further modulate elastin homeostasis in AAA pathobiology. Clinically, biomarkers of elastin turnover and elastin-targeted molecular imaging techniques are emerging as tools for risk stratification. Therapeutically, novel strategies aimed at stabilizing elastin fibers, enhancing cross-linking, or delivering drugs directly to sites of elastin damage have shown promise in preclinical models and early translational studies. In parallel, regenerative approaches employing stem cells, exosomes, and bioengineered elastin scaffolds are under development to restore structural integrity. Collectively, these advances underscore the pivotal roles of elastin not only as a structural determinant of aneurysm development but also as a diagnostic and therapeutic target. This review summarizes and integrates recent discoveries on elastin biology in AAA, with a particular emphasis on molecular mechanisms of elastin degradation and the translational potential of elastin-centered interventions for the prevention and treatment of AAA.

## 1. Clinical Background, Epidemiology, and Current Management of AAA

Abdominal aortic aneurysms (AAAs) are permanent dilations of the abdominal aorta that progressively undermine the structural integrity of the vessel wall and often remain clinically silent until catastrophic rupture, which carries a 70–85% case-fatality rate [1,2,3]. Because most AAAs are asymptomatic, abdominal ultrasound (US) and duplex ultrasonography (DUS) are recommended as first-line, guideline-endorsed tools for detection, surveillance, and population-based screening among high-risk groups (e.g., older male smokers) [4]. Operationally, an abdominal aortic diameter ≥ 3.0 cm—typically >2 standard deviations above the mean for men—meets the conventional threshold for an aneurysmal aorta [5]. To account for inter-individual and segmental differences, others propose defining AAA as a maximum infrarenal diameter > 1.5 × the expected normal infrarenal (or suprarenal) diameter, thereby normalizing for adjacent aortic size and the specific measurement site [6]. Epidemiologically, AAA remains a substantial public health burden (e.g., 4903 AAA-related deaths in the United States in 2018; crude rate 1.5 per 100,000), with risk concentrated among individuals ≥ 65 years, current or former smokers, men, and those with family history, chronic obstructive pulmonary disease (COPD), atherosclerotic cardiovascular disease, hypertension, or hyperlipidemia [7]. Although AAAs frequently coexist with intraluminal atheroma, atherosclerosis is neither necessary nor sufficient for aneurysm formation, and AAAs may occur without it [8,9]. Diabetes mellitus, paradoxically, is associated with lower AAA prevalence and slower growth. Large population-based analyses and meta-analyses consistently report reduced AAA incidence and ≈30–40% slower expansion in people with diabetes compared with non-diabetics under surveillance. Moreover, men develop AAA more frequently and at younger ages than women, whereas women—though less commonly affected—face faster growth and higher rupture risk once aneurysms are present; multiple lines of evidence indicate that estrogen signaling is protective. In angiotensin II (Ang II)-driven murine AAA, exogenous 17β-estradiol lowers aneurysm incidence and severity and blunts progression of established lesions; conversely, ovariectomy increases AAA incidence. Human/experimental data show higher aortic ERα expression in females that associates with fewer aneurysms.

Aneurysmal dilation progresses over time [10], and larger aneurysms expand faster than smaller ones [11,12,13], a behavior tightly linked to loss of aortic elasticity and mechanical resilience. This acceleration reflects degradation of elastin, the load-bearing elastic fiber network and apoptosis/phenotypic modulation of vascular smooth muscle cells (VSMCs), which together diminish wall recoil, impair matrix repair capacity, and amplify mural stress, thereby increasing the propensity for maladaptive remodeling, continued sac expansion, and ultimately dissection and/or rupture. Despite decades of investigation, no pharmacotherapy has conclusively shown efficacy in halting AAA growth. Clinical trials and translational studies have explored doxycycline as a broad MMP modulator [14,15,16,17], beta-blockers targeting hemodynamic load [18,19,20,21], ACE inhibitors with anti-inflammatory/vascular effects [22,23,24], and statins with pleiotropic vascular benefits [25,26,27,28], yet results remain inconclusive, and additional investigations are ongoing [28,29]. Consequently, management for anatomically suitable or rapidly enlarging AAAs relies on endovascular aneurysm repair (EVAR) with a stent graft [30,31,32] or open surgical replacement of the diseased segment with a prosthetic graft [33,34]. However, both interventions entail procedural risk and may be unsuitable for patients with prohibitive operative risk or unfavorable anatomy, underscoring a persistent unmet need for disease-modifying medical therapies that stabilize the aortic wall, preserve elastin integrity, and delay or obviate invasive repair [35,36].

## 2. Elastin Structure and Cross-Linking in the Aortic Wall

The remarkable elastic properties of the aorta are supported by a dense network of elastic fibers within the extracellular matrix (ECM) [37,38]. At the core of these fibers is the elastin, the predominant structural ECM component of the aortic media, encoded by a single gene in mammals and secreted by VSMCs as a 60–70 kDa soluble monomer, tropoelastin [39]. Tropoelastin contains two broad classes of peptide domains: lysine-rich amino acids that serve as substrates for covalent cross-linking, and hydrophobic amino acids rich in glycine and proline that drive self-association and endow the mature polymer with elastic recoil. With the assistance of microfibrillar scaffolds (principally fibrillins) and accessory proteins (e.g., fibulins), tropoelastin assembles on the cell surface into extensible elastic fibers integrated with microfibrils [39]. Subsequent coacervation concentrates hydrophobic segments into liquid-like assemblies that align on the microfibrillar template. Lysyl oxidase-mediated oxidation of lysine residues then generates allysines that condense into desmosine/isodesmosine cross-links, converting these nascent aggregates into insoluble, load-bearing elastic fibers. Fibulins and other accessory proteins coordinate deposition and bundling with fibrillin-rich microfibrils, yielding mature elastic lamellae that alternate with VSMC layers in the aortic media and confer durable elastic recoil across the cardiac cycle [40,41]. Notably, the infrarenal abdominal aorta contains fewer elastic lamellae and a lower elastin-to-collagen ratio than the thoracic aorta, a regional difference thought to contribute to the predilection of AAAs for this segment.

Elastin is insoluble, hydrophobic, heat-stable, and highly cross-linked, properties that underline its extraordinary durability and resistance to proteolysis [42,43]. For a long time, elastin has been considered as an inert protein formed during the development and lasting for almost the whole life of the subjects. This is due to the life span of elastin is estimated to be 80 years using aspartic acid racemization and ^14^C turnover methods [44]. However, the subsequent studies demonstrate that elastin is a subject of many insults and can be degraded [45,46,47]. And due to extremely low turnover rate, this degradation is practically an irreversible and irreparable process [48,49,50,51,52,53]. Consistent with these physical properties, elastin’s amino-acid composition is unusual—approximately one-third glycine, ~10–13% proline, and >40% other hydrophobic residues—combined with a high density of intermolecular cross-links that render the mature polymer refractory to enzymatic degradation [54]. The hydrophobic domains drive coacervation, a temperature-dependent self-assembly of tropoelastin into condensed droplets that facilitate an ordered alignment of monomers and subsequent cross-linking [55,56,57].

**Figure 1:**
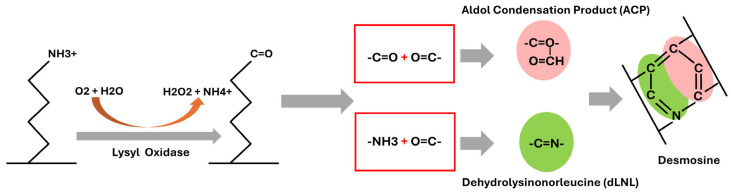
Mechanism of elastin cross-linking and desmosine formation. There are two major bifunctional cross-links in elastin: dehydrolysinonorleucine (dLNL), formed through the condensation of one residue of allysine and one of lysine, and aldol condensation product (ACP) formed through the association of two allysine residues. ACP and dLNL then condense to form the cross-link desmosine.

Tropoelastin is encoded by a single gene localized to chromosome 7q11.23 in humans [58]. Elastin expression in most tissues occurs over a narrow window of development, beginning in mid-gestation and continuing at high levels through the postnatal period [59,60]. In aorta, expression decreases rapidly when the physiological rise in blood pressure stabilizes postnatally, and there is minimal elastin synthesis in any tissue in the adult animal [61,62,63]. This explains why damage to elastic fibers during the adult period is so detrimental and why the elastin protein must have a long half-life. A critical feature of the elastic fiber, crucial to its proper function, is the extensive extracellular cross-linking of tropoelastin mediated by the enzyme protein called lysine-6-oxidase (LOX), which oxidizes selective lysine residues in peptide linkage to a-aminoadipic d-semialdehyde [64]. Cross-linking of elastin monomers is initiated by the oxidative deamination of lysine side chains by LOX in a reaction that consumes molecular oxygen and releases ammonia. The aldehyde that is formed can condense with another modified side chain aldehyde to form the bivalent aldol condensation product (ACP) cross-link. Reaction with the amine of an unmodified side chain through a Shiff base reaction produces dehydrolysinonorleucine (dLNL). ACP and dLNL then condense to form the tetrafunctional cross-link desmosine (Figure 1). Degeneration of elastic laminas has been observed in LOX knockout (KO) mice [65] and heterozygous blotchy mouse [66], and aneurysmal changes were observed in large arteries [65,66]. LOX activity can be pharmacologically inhibited by beta-aminopropionitrile (BAPN) [67]. Combination of elastic lamina degeneration by LOX inhibition and hypertension causes thoracic and abdominal aortic aneurysms formations [68].

## 3. Molecular Mechanism Involved in Elastin Degradation in AAA

**Figure 2:**
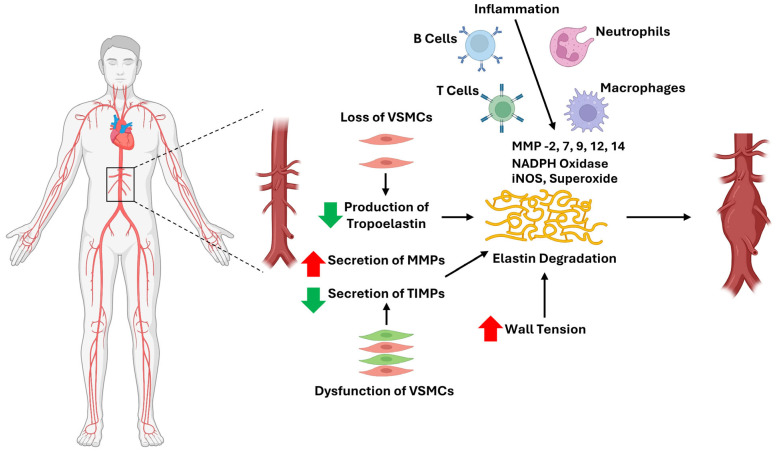
Mechanisms and mediators of abdominal aortic aneurysm formation and progression. The main cellular mechanisms and molecular mediators involved in the formation and progression of abdominal aortic aneurysms are schematically depicted. Abbreviations: VSMC, vascular smooth muscle cell; MMP, matrix metalloproteinase; NADPH, nicotinamide adenine dinucleotide phosphate; iNOS, inducible nitric oxide synthase; TIMP, tissue inhibitor of matrix metalloproteinase.

AAA is caused by the interaction of environmental [69], genetic [70,71], and biochemical factors [72,73]. The loss of VSMCs and degeneration of the aortic media ECM have long been identified as hallmark of AAA pathology (Figure 2). Loss of VSMC causes less production of tropoelastin, therefore impairs the turnover and repair of elastin damages. Additionally, dysfunctional VSMC produces more MMP-2 and less TIMPs and thus accelerates elastin degradation (Figure 2). Infiltrating immune cells, mainly macrophages and neutrophils, can produce a large amount of reactive oxygen species (ROS) and different MMPs [46,47,74,75,76,77]. Biomechanical wall stress also contributes to elastin degradation and therefore aggravates the diseased state [78] (Figure 2). The loss of structural integrity due to elastin degradation is key to aortic dilation and rupture. However, the turnover rate of elastin in vivo is very low. Studies using sensitive immunological techniques to measure elastin peptides in the blood or desmosine cross-links excreted in the urine suggest that less than 1% of the total body elastin pool turns over in a year [79]. Extremely low turnover rate of elastin results in constant loss of elastic lamina.

A central question is whether elastin degradation is an etiologic driver of AAA or a secondary outcome of other processes. In truth, it functions as both. A direct and localized injury to elastic lamellae is sufficient to launch aneurysmal remodeling. The classic intraluminal elastase perfusion or topical/periadventitial elastase model produces segmental infrarenal dilatation with near-total loss of medial elastin, whereas saline-perfused controls do not dilate, an observation that has been reproduced across species and protocols and remains foundational in the field [80,81]. The same principle holds true when the elastic scaffold is weakened upstream at the level of cross-linking. Genetic LOX disruption leads to perinatal aortic aneurysm/rupture in mice, establishing that intact elastin cross-linking is indispensable for aortic integrity. Pharmacologic cross-link blockade with BAPN reliably “primes” the aorta for dilation in elastase or Ang II infusion models, with aneurysm incidence and growth scaling with elastase dose and the degree/duration of cross-link inhibition [65,82]. These causal manipulations show that elastin damage can be an initiating factor. They also reveal a characteristic latency between the initial insult and overt dilatation, implying that inflammation and secondary proteolysis are recruited downstream of the primary elastin breakage to complete the aneurysm phenotype [83]. Mechanistic refinements reinforce this axis: increasing the pressure of elastase perfusion (more severe elastolysis) increases aneurysm formation and size, and combining topical elastase with BAPN produces “true” infrarenal AAAs that recapitulate human disease behavior, again tying the intensity of elastin injury to disease penetrance and growth [84,85]. Convergent human data echo these experimental relationships: circulating desmosine/elastin–breakdown products associate with AAA severity and predict adverse outcomes and future expansion, providing a measurable link between ongoing elastolysis and subsequent growth dynamics [86]. On the other hand, elastin break is also a consequence. Once the elastin-injured niche exists, a proteolytic–inflammatory program accelerates further elastin loss. Genetic “loss-of-function” experiments demonstrate that removing key elastases diminishes aneurysm formation or slows progression. Mice lacking MMP-9 or MMP-2 are protected in elastase/CaCl_2_ models with preservation of matrix; deficiency or inhibition of cysteine cathepsins (K and S) likewise reduces elastin fragmentation and AAA burden; and deleting neutrophil elastase/proteinase-3 also suppresses AAAs and dampens downstream NET-driven inflammation [87,88,89,90].

Importantly, elastin destruction does not merely mark damage; it amplifies it. Elastin-derived peptides generated by proteolysis act as chemoattractants via the 67-kDa elastin-binding protein and reprogram macrophages toward a proinflammatory M1 state. Neutralizing these peptides curbs cytokine induction, MMP activity, and dilation in vivo, providing causal evidence for a feed-forward loop in which elastolysis begets inflammation that, in turn, begets further elastolysis [91,92]. The macroscopic mechanics of the wall then compound the biology: loss of the low-strain elastic scaffold shifts load to collagen at smaller strains, raises peak wall stress for a given diameter/pressure, and is associated with faster enlargement and higher rupture risk in finite-element analyses—biomechanical signatures that are consistently observed as elastin content falls [93]. Finally, the loop’s reversibility underscores causality. Stabilizing or protecting elastic fibers with pentagalloyl glucose (PGG) after injury, via local or targeted systemic delivery, reduces MMP activity, preserves elastin, and attenuates AAA growth in rats, with systematic reviews noting benefit across several animal studies (albeit with heterogeneous methods and quality), which reinforces the concept that interrupting elastin loss can decelerate the disease [94,95,96].

### 3.1. Protease-Driven Elastinolysis in AAA

AAAs are characterized by the presence of matrix-degrading enzymes such as MMPs [40,69,97,98]. An imbalance between active MMPs and their inhibitors including TIMPs are responsible for most of the changes in the medial matrix. In the normal aorta, endothelial cells, VSMCs, and adventitial fibroblasts are responsible for MMP production. In the setting of AAA, inflammatory cells serve as additional sources of MMPs. Thus far, 23 different MMPs have been described in humans and are divided into archetypal, matrilysins, gelatinases, and furin-activated MMPs. However, only five matrix metalloproteinases have been shown to degrade elastin and its precursor tropoelastin, namely MMP-2, -7, -9, -14 [46,47,74,75,76,77]. Functionally, this remodeling replaces a resilient, energy-storing vessel with one that is stiffer yet mechanically weaker, predisposed to progressive dilation and rupture.

MMP-2 (gelatinase A) degrades elastin and is expressed constitutively in VSMCs, fibroblasts, and macrophages. Ang II and CaCl_2_ augment MMP-2 activity in the abdominal aorta. In vivo studies reveal that MMP-2 systemic KO had no impact on Ang II–induced AAAs but was protective of CaCl_2_-induced AAAs, likely due to compensatory mechanisms by other MMPs. Indeed, MMP-9 (gelatinase B) is constitutively produced by fibroblasts, VSMCs and by infiltrating adventitial macrophages during AAA formation.

MMP-9 protein level in the aortic wall and plasma is significantly higher in patients with AAAs, as well in patients with a luminal thrombus. MMP-9 KO mice are resistant to elastase-induced AAAs. Infusion of wild-type macrophages into MMP-9 KO mice leads to reconstitution of AAA, suggesting macrophage-derived MMP-9 is crucial for AAA formation.

MMP-12 is a metalloelastase degrading both soluble and insoluble elastin. MMP-12 is mainly produced and secreted by macrophages and responsible for approximately 95% of the macrophage-mediated elastin degradation [99,100]. The role of MMP-12 in AAA is not entirely clear. MMP-12 knockout mice exhibit a small increase in aortic diameter compared with the wild type (WT) mice during AAA induction [101]. However, studies using mice genetically deficient in both MMP-9 and MMP-12 show that MMP-12 may not be directly involved in AAA formation although it may facilitate other MMPs like MMP9 in matrix degradation [83].

MMP-14 is a membrane type 1 MMP produced by infiltrating macrophages and VSMCs in the aortic wall [102,103]. High MMP-14 mRNA and protein expression levels are found in human AAAs. In murine CaCl2-induced AAAs, macrophage-derived MMP-14 plays a crucial role in elastin degradation in the tunica media and adventitia, leading to AAA formation [104]. MMP-14 is also involved in the activation of proMMP-2 by binding to TIMP2 [105].

MMP-7 also degrades elastin and plays a role in lung defense mechanisms [106,107]. MMP-7 is significantly increased in plasma and aneurysm tissues of patients with AAA associated with hypertension [108]. MMP-7 deficient mice in ApoE background show a higher VSMC content compared to WT mice [109]. It appears that MMP-7 promotes VSMC apoptosis by cleaving N-cadherin [110].

MMPs are regulated at several different levels such as gene expression, activation, and cellular inhibition of activity by TIMPs, especially TIMP-1 and TIMP-2. TIMP1 and TIMP2 are secretory proteins that inhibit the proteolytic activity of MMPs by forming noncovalent 1:1 stoichiometric complex and regulate the balance of matrix remodeling during degradation of extracellular matrix [111]. An animal study has shown that the increased ratios of MMP-9/TIMP-1 and MMP-2/TIMP-2 in aneurysmal walls lead to worse progression of AAAs [112].

### 3.2. VSMC Loss–Driven Elastin Depletion

VSMC apoptosis and aortic media degeneration have long been considered as the hallmark of AAA pathology. Inflammation, ROS production, and endoplasmic reticulum (ER) stress are all associated with VSMC apoptosis in AAA [113,114,115,116,117,118,119,120,121,122,123]. Inflammation furnishes a cytokine milieu (e.g., TNF-α, IL-1β, IFN-γ) that activates death signaling and pro-apoptotic transcriptional programs in VSMCs [113,114,115,116,117,118,119,120,121,122,123]. ROS arises from Nicotinamide Adenine Dinucleotide Phosphate (NADPH) oxidases, myeloperoxidase-rich leukocyte infiltrates, dysfunctional mitochondria, and iron/hemoprotein fluxes within the intraluminal thrombus. These oxidants damage DNA, lipids, and proteins, and amplify NF-κB/AP-1 signaling, which sensitize VSMCs to cell death [113,114,115,116,117,118,119,120,121,122,123]. In parallel, ER stress, driven by sustained inflammatory load, disturbed calcium homeostasis, proteotoxicity, and oxidant injury, activates the unfolded protein response. If unresolved, this response shifts from adaptive to pro-apoptotic, converging on CHOP and caspase pathways that execute VSMC apoptosis [113,114,115,116,117,118,119,120,121,122,123]. The superimposed mechanical strain in the expanding sac and protease-rich microenvironments further destabilize cytoskeletal-matrix adhesions and prime VSMCs for death, completing a local ecosystem that consistently favors VSMC attrition [113,114,115,116,117,118,119,120,121,122,123].

The consequences of VSMC loss extend beyond simple cell depletion. First, VSMC apoptosis removes the principal source of tropoelastin and elastin-organizing machinery in the media. Surviving cells often adopt a synthetic or proinflammatory phenotype with decreased elastin production, causing the capacity to replenish or reorganize elastic lamellae sharply curtailed. Second, with fewer VSMCs, cross-link maturation of newly deposited tropoelastin, e.g., via LOX-dependent desmosine/isodesmosine formation, is also compromised, making nascent fibers more vulnerable to enzymatic and mechanical failure. Third, apoptotic bodies and uncleared debris serve as danger-associated molecular patterns that perpetuate leukocyte recruitment and protease induction, deepening elastinolysis and ECM attrition. Collectively, these processes convert the media from a self-repairing, energy-storing layer into a patchwork of fragmented elastin, disorganized collagen, and depleted cells, an architecture that is stiffer yet mechanically weaker, and thus progressively predisposed aorta to expansion and rupture [39,105,107].

An additional layer of vulnerability arises from regional VSMC ontogeny. Most AAAs occur below the renal arteries, a distribution that may reflect intrinsic differences in VSMC developmental origin and baseline gene programs. The distal abdominal aortic VSMCs derive from mesoderm, whereas thoracic aortic VSMCs largely arise from neural crest [124]. These lineage distinctions are associated with region-specific differences in matrix synthesis, stress responsiveness, and inflammatory signaling, potentially explaining why infrarenal VSMCs are less resilient to chronic oxidative/inflammatory stress and mechanical load [125]. In this context, loss of mesoderm-derived VSMCs in the infrarenal segment disproportionately reduces local elastin synthesis and maintenance, accelerating elastic lamellar fragmentation and cementing the VSMC-elastin axis as a non-proteolytic pathway to elastin loss that operates alongside protease-mediated degradation.

### 3.3. Inflammatory Orchestration of Elastinolysis in AAA

A defining feature of AAA initiation and progression is a spatiotemporally organized inflammatory response within the aortic wall that both triggers and sustains elastin breakdown. Neutrophil infiltration occurs early after injury and is typically transient, yet it is functionally decisive. In the elastase model, neutrophil-neutralizing antibodies attenuate aneurysm expansion, directly implicating neutrophils in disease initiation [90]. Mechanistically, neutrophils deliver a concentrated burst of ROS via NADPH oxidases and myeloperoxidase (MPO) [126,127,128]. These oxidants (e.g., superoxide, hypochlorous acid) damage elastic fibers, oxidatively inactivate endogenous protease inhibitors, and prime MMP activity, collectively tipping the local environment toward elastinolysis [128]. Concomitantly, neutrophil degranulation releases serine proteases and creates a protease-rich pericellular niche that weakens elastic lamellae and exposes additional matrix targets to cleavage, amplifying the impact of even a short-lived neutrophil presence.

As acute inflammation transitions to chronicity, macrophage infiltration becomes one of the most consistent pathological events in human and experimental AAA lesions [129,130,131]. Recruited monocyte-derived macrophages populate the media and adventitia where they produce MMPs (notably MMP-9), elaborate cytokines and chemokines that perpetuate leukocyte recruitment, and phagocytose cellular/matrix debris [129,130,131]. Through these actions, macrophages sustain and amplify the proteolytic milieu initiated by neutrophils. Macrophage-derived MMPs directly cleave elastin and the associated microfibrils, while inflammatory signaling further upregulates protease expression in both infiltrating leukocytes and resident vascular cells [129,130,131]. The net effect is a feed-forward inflammatory-proteolytic axis: early neutrophil ROS and proteases weaken the elastic scaffold and disable inhibitory brakes, after which macrophage-dominated MMP production and cytokine signaling that maintain high-grade elastin degradation over time. This coordinated leukocyte succession transforms the aortic wall from a resilient, energy-storing conduit into a stiffer and mechanically weaker artery, accelerating sac expansion and increasing the risk of rupture [129,130,131].

### 3.4. Links Between AAA Risk Factors and Elastin Degradation

Advanced age, male sex, and cigarette smoking converge mechanistically on the elastic scaffold of the infrarenal aorta to accelerate elastin degeneration in distinct but complementary ways. Aging progressively degrades elastic lamellae through decades of cyclic mechanical fatigue and low-grade enzymatic injury. Histologic series in humans demonstrate age-correlated elastin fragmentation and fibrosis. Comprehensive reviews show that mechanical load is consequently transferred from compliant elastin to stiffer collagen and that accumulation of nonenzymatic cross-links with age further stiffens aortic wall and diminishes recoil, promoting dilatation under physiologic pressure [132,133]. Smoking is the dominant modifiable risk factor for AAA and displays a strong dose–response relationship with disease incidence. Meta-analysis across prospective cohorts reports ≈ 5-fold higher risk in current smokers (and ≈2-fold in former smokers) versus never-smokers, consistent with a causal role for tobacco exposure. Mechanistically, chronic smoke or nicotine exposure fosters macrophage and neutrophil infiltration and upregulates elastolytic programs in the aortic wall. In mice, sustained nicotine exposure increases aortic MMP activity, induces marked elastin fiber thinning/fragmentation, and produces stiffness segmentation, while pharmacologic MMP inhibition in this setting reduces MMP activity, limits elastin destruction, and prevents stiffness progression, which link smoking directly to elastinolysis rather than merely to inflammation [134,135,136].

Sex hormone also modulates this axis: estrogen signaling is protective for the elastic matrix. In AngII-driven murine AAA, exogenous 17β-estradiol blunts progression of established lesions. Conversely, ovariectomy increases elastin breaks. Human/experimental data show higher aortic ERα expression in females that associates with lower MMP activity, together supporting a model in which female sex hormones dampen protease- and oxidative-stress–mediated elastin degradation. These epidemiologic and mechanistic strands (aging, smoking, and sex) converge on a final common pathway—accelerated elastin loss in the medial layer—through which age-related elastic fiber fatigue, tobacco-triggered proteolysis/oxidation, and androgen-biased (or estrogen-deficient) inflammatory signaling collectively weaken the human infrarenal aorta and foster aneurysm formation and expansion [137,138].

Multiple mechanistic observations explain the protection of diabetes at the level of extracellular matrix architecture. A leading mechanistic explanation is chronic hyperglycemia-driven formation of advanced glycation end-products (AGEs) that create extensive intermolecular cross-links within collagen and elastin. These glycation-mediated cross-links stiffen the matrix and render it more resistant to proteolytic attack, thereby impeding elastin fiber thinning and medial delamination that ordinarily fuel aneurysmal enlargement. Human tissue studies show higher levels of cross-linking AGEs (e.g., pentosidine) in diabetics with AAA and link these to slower growth, while interventional work indicates that disrupting or preventing AGE cross-linking accelerates elastin degradation and smooth-muscle injury—evidence that the cross-links themselves are protective in the aneurysm setting. Beyond intrinsic matrix effects, several antidiabetic agents appear to reinforce this protection by dampening inflammation and protease activity. Observational meta-analyses associate metformin prescription with slower AAA growth and fewer clinically relevant events. Preclinical studies show that metformin and other glucose-lowering agents (e.g., DPP-4 inhibition with sitagliptin; PPAR-γ agonism with pioglitazone) can attenuate Ang II- or elastase-induced AAAs by reducing macrophage chemokines, MMP expression/activity, and oxidative stress in the aortic wall [139,140,141,142,143,144,145,146].

## 4. Current and Potential Novel AAA Therapy Targeting Elastin

Because AAA pathogenesis is multifactorial and self-reinforcing, single-node interventions (e.g., broad anti-inflammatories or solitary MMP blockades) have repeatedly struggled to yield durable benefits in vivo. The long-term, spatially adequate suppression of local inflammation and protease activity is difficult to achieve and, when intensified, can be accompanied by adverse effects. Although genetic and pharmacologic inhibition of MMPs, augmentation of endogenous inhibitors (TIMPs), and upstream anti-inflammatory strategies have shown signal in experimental systems, none has yet matured into a standard clinical therapy. A key reason is the network redundancy: multiple enzymes, cell types, and injury pathways act with temporal and spatial specificity across AAA initiation and growth. Therefore, knocking down one arm of the cascade often diverts flux to others. In this context, therapeutic strategies that stabilize or restore the elastin scaffold itself, the common target of many destructive pathways, are conceptually attractive. Rather than chasing every upstream driver, directly preserving elastin architecture offers a tractable way to maintain wall mechanics, blunt positive feedback (protease induction, inflammatory recruitment), and potentially re-set the biology toward quiescence. Consistent with this logic, a growing body of preclinical work has introduced elastin-centric therapies that show promising effects on aneurysm growth, wall composition, and inflammatory tone.

### 4.1. Elastin Stabilization (“Tanning” the Elastic Lamellae)

One prominent approach is small-molecule stabilization of existing elastic fibers to resist proteolysis and fatigue. Pentagalloyl glucose (PGG), a polyphenolic molecule with high affinity for elastin hydrophobic domains, binds non-covalently to exposed elastin, shielding cleavage-prone motifs, reducing water ingress into hydrophobic repeats, and sterically limiting protease access [147]. In multiple AAA models, PGG has preserved elastic lamellae, reduced inflammatory infiltration, and slowed or prevented sac expansion. [94,148]. Localized periadventitial delivery of non-cytotoxic PGG early after injury inhibits elastin degeneration and attenuates AAA growth, indicating that targeted wall exposure can modify disease trajectory even without systemic drug levels [94]. Extending this concept, PGG-loaded nanoparticles have been engineered to home to the aneurysmal wall, where controlled release restores degraded elastin ultrastructure, reduces MMP activity, and diminishes leukocyte infiltration. Notably, oral administration of PGG nanoparticles has regressed established elastase-induced AAAs in preclinical studies, suggesting that systemic routes can achieve therapeutically meaningful aortic exposure when coupled with appropriate carriers [149]. Mechanistically, PGG effects likely arise from a triad: (i) direct fiber stabilization which reduces elastase susceptibility, (ii) secondary anti-inflammatory actions, i.e., less Damage-Associated Molecular Patterns release from matrix breakdown due to lower cytokine/MMP induction, and (iii) favorable reparative remodeling by providing a protected scaffold onto which new matrix can organize [117,118,119,120]. While PGG is an example, this “elastin-tanning” principle may extend to other elastin-binding polyphenols, tailoring small molecules with improved pharmacokinetics and tissue selectivity.

### 4.2. Elastin-Targeted Drug Delivery (Active Targeting to Sites of Damage)

Because the aneurysmal wall is characterized by patchy elastin fragmentation, elastin itself becomes a surface epitope for active targeting. Degraded/fragmented elastin exposes cryptic motifs that can be recognized by anti-elastin antibodies, enabling drug carriers to bind specifically to diseased wall segments while sparing healthy vasculature. In rats, nanoparticles conjugated with anti-elastin antibodies accumulate at the aneurysm site after systemic injection, demonstrating in vivo targeting feasibility [149,150]. Loading these NPs with batimastat (BB-94; a potent MMP inhibitor) or rapamycin has prevented aneurysmal growth in elastase- and CaCl_2_-based AAA models. This approach achieves local therapeutic concentrations with orders-of-magnitude lower systemic exposure than free drug; thereby minimizing off-target toxicity while maximizing on-target protease suppression and anti-inflammatory effects [150]. Conceptually, elastin-targeted carriers can be modular, i.e., the same targeting shell can deliver (i) protease inhibitors (batimastat, doxycycline analogs), (ii) redox modulators, (iii) pro-elastogenic cues (e.g., agents that upregulate LOX activity or tropoelastin expression), and (iv) theragnostic payloads (imaging agents co-delivered to map drug engagement). This platform is well-suited to heterogeneous diseases. Drug is concentrated where elastin damage (and thus risk) is the greatest, enabling precision dosing of the highest-need segments. Practically, elastin-targeted systems must optimize affinity vs. penetration (tight binding can impede deep wall distribution), carrier size and charge (governing biodistribution and reticuloendothelial system uptake), and release kinetics (sustained exposure vs. burst). The proof-of-concept efficacy in rodents appears to be robust [120,121].

## 5. Summary

This review synergizes structural, mechanistic, and translational insights to argue that elastin is the central determinant of AAA stability, both as the principal load-bearing element of the aortic media and as a convergence point for inflammatory, proteolytic, and biomechanical injury. The structural scarcity and low turnover of elastin in the infrarenal aorta create baseline vulnerability. The inflammation-driven protease cascades (MMP-2, -7, -9, -12, -14) and oxidative stress initiate and expand elastin fragmentation. VSMC loss suppresses elastin synthesis and cross-link maturation, starving repair. And the mechanical stress and calcific remodeling stiffen yet weaken the wall, accelerating dilation. From this vantage, protecting and strategically rebuilding the elastin scaffold becomes a clear path to correct disease modification. The early preclinical successes with PGG “tanning” and elastin-targeted nanoparticles bring the goal within reach. Therefore, the precision and stage-specific trials that pair elastin-focused therapeutics with mechanistically aligned biomarkers/imaging and clinically meaningful endpoints (growth rate, biomechanical surrogates, intervention-free survival) may be promising. If realized, this approach could move AAA care beyond the current paradigm of “monitor until repair” toward active, anatomy-guided therapy that preserves the elastin architecture foundational to aortic stability.

## Data Availability

No new data were created or analyzed in this study.
